# Vector competence of sterile male *Glossina fuscipes fuscipes* for *Trypanosoma brucei brucei*: implications for the implementation of the sterile insect technique in a sleeping sickness focus in Chad

**DOI:** 10.1186/s13071-023-05721-4

**Published:** 2023-03-22

**Authors:** Mahamat Hissene Mahamat, Adeline Ségard, Jean-Baptiste Rayaisse, Rafael Argiles-Herrero, Andrew Gordon Parker, Philippe Solano, Adly Mohamed Mohamed Abd-Alla, Jérémy Bouyer, Sophie Ravel

**Affiliations:** 1 Institut de Recherche en Elevage pour le Développement (IRED), Ndjaména, Chad; 2 Université de Montpellier, Cirad, IRD, Intertryp, Montpellier, France; 3 Centre International de Recherche-Développement sur l’Elevage en Zone Subhumide (CIRDES), Bobo-Dioulasso, Burkina Faso; 4 Joint Food and Agriculture Organization of the United Nations/International Atomic Energy Agency Centre of Nuclear Techniques in Food and Agriculture, Insect Pest Control Sub-programme, A-1400 Vienna, Austria; 5 Université de Montpellier, Cirad, INRAE, ASTRE, Montpellier, France

**Keywords:** Tsetse, Isometamidium, Trypanocide, Trypanosoma brucei gambiense

## Abstract

**Background:**

Human African trypanosomiasis (HAT) is a neglected tropical disease caused by *Trypanosoma brucei gambiense* transmitted by tsetse flies in sub-Saharan West Africa. In southern Chad the most active and persistent focus is the Mandoul focus, with 98% of the reported human cases, and where African animal trypanosomosis (AAT) is also present. Recently, a control project to eliminate tsetse flies (*Glossina fuscipes fuscipes*) in this focus using the sterile insect technique (SIT) was initiated. However, the release of large numbers of sterile males of *G. f. fuscipes* might result in a potential temporary increase in transmission of trypanosomes since male tsetse flies are also able to transmit the parasite. The objective of this work was therefore to experimentally assess the vector competence of sterile males treated with isometamidium for *Trypanosoma brucei brucei*.

**Methods:**

An experimental infection was set up in the laboratory, mimicking field conditions: the same tsetse species that is present in Mandoul was used. A *T. b. brucei* strain close to *T. b. gambiense* was used, and the ability of the sterile male tsetse flies fed on blood with and without a trypanocide to acquire and transmit trypanosomes was measured.

**Results:**

Only 2% of the experimentally infected flies developed an immature infection (midgut) while none of the flies developed a metacyclic infection of *T. b. brucei* in the salivary glands. We did not observe any effect of the trypanocide used (isometamidium chloride at 100 mg/l) on the development of infection in the flies.

**Conclusions:**

Our results indicate that sterile males of the tested strain of *G. f. fuscipes* were unable to cyclically transmit *T. b. brucei* and might even be refractory to the infection. The data of the research indicate that the risk of cyclical transmission of *T. brucei* by sterile male *G. f. fuscipes* of the strain colonized at IAEA for almost 40 years appears to be small.

**Graphical Abstract:**

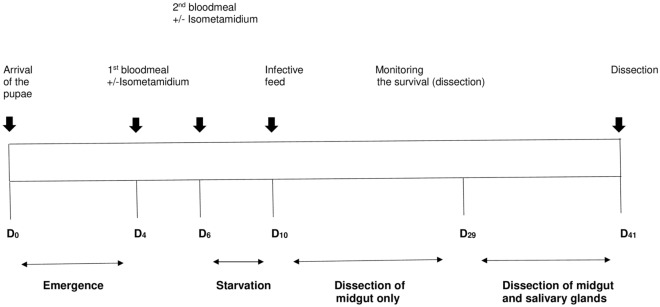

**Supplementary Information:**

The online version contains supplementary material available at 10.1186/s13071-023-05721-4.

## Background

Human African trypanosomiasis (HAT), or sleeping sickness, is an endemic neglected tropical disease in sub-Saharan Africa caused by subspecies of *Trypanosoma brucei* transmitted by tsetse flies (*Glossina*). The World Health Organization (WHO) aims to reach elimination as interruption of transmission of Gambiense HAT by 2030 [1].

The Mandoul focus, located in the Logone Oriental part of Southern Chad, is a historical g-HAT focus [2] showing the highest transmission rate in the country in the last decades. It is an agro-pastoral area covering > 520 km^2^ with fertile land because of the proximity of a river, where there are many cattle, sheep, goats and pigs [2] and where African animal trypanosomosis (AAT) is also present [3, 4]. *Glossina fuscipes fuscipes* was found as the only cyclical vector of trypanosomes in this area [3], with an apparent density of 0 to 26 tsetse flies per trap per day at the end of 2013 [5]. After 4 years of tsetse control using tiny insecticide-impregnated targets from 2014 to 2017, a 99% reduction was observed compared to the initial tsetse densities before control combined with a strong decrease in the prevalence of HAT [5]. To sustain this interruption of transmission, local eradication of the vector appears a promising strategy. A Chadian project has thus been initiated to eliminate tsetse flies from this area using the sterile insect technique (SIT). This approach has been previously implemented successfully against *Glossina* *austeni* in Zanzibar and against *Glossina palpalis gambiensis* in Senegal, where AAT occurs [6–8]. However, since this will be the first time that SIT will be used in an area with the human form of the disease, it is important to know whether the released sterile males may potentially be able to transmit trypanosomes to humans.

For this purpose, in the laboratory we evaluated the ability of the sterile males from a mass-reared strain of *G. f. fuscipes*, intended for use in Chad, to acquire and transmit trypanosomes under conditions where tsetse flies receive trypanocide prior to the infective blood meal, as in other SIT programs [8].

## Methods

### Tsetse flies

The *G. f. fuscipes* strain used here originated from the Central African Republic and has been reared at the Insect Pest Control Laboratory (IPCL), Joint FAO/IAEA Centre for Nuclear Techniques in Food and Agriculture insectary in Seibersdorf, Austria, since 1986. Sterile males were irradiated at the pupal stage with 120 Gy, decreasing fertility by > 95% [9]. The tsetse pupae were irradiated in air at the IPCL, Seibersdorf, Austria, using a Gammacell® 220 (MDS Nordion Ltd., Ottawa, Canada) ^60^Co irradiator. The dose rate was measured by alanine dosimetry as 2.144 Gy·s^−1^ on 3 March 2015 with an expanded uncertainty (k = 2) of 3.2%. Sterile pupae (one batch/one shipment) were transported to the insectary in Montpellier under chilled conditions (at 10 °C, see [10] for more details) with Fedex® transport over 24 or 48 h. Then, the newly emerged sterile males were put in Roubaud cages (13 × 8 × 5 cm) in groups of 25 adults and maintained at 25 ± 1 °C, 80 ± 5% rH and 12:12 light/dark photoperiod.

Sterile males were separated into two batches (1 and 2). At day 4 and day 6 after receipt, batch 1 received a preheated blood meal for 30 min away from light on an in vitro silicon membrane system maintained at 37 °C, using defibrinated sheep blood collected aseptically and previously frozen at − 20 °C and supplemented with isometamidium chloride at 100 mg/l as previously described by Van den Bossche et al. [11]. Batch 2 was offered two blood meals without the trypanocide.

### Mouse infection with *Trypanosoma brucei brucei*

A stabilate of infected mouse blood with *T. b. brucei* J10 WT [12] was thawed and passaged via intraperitoneal injection into two immunosuppressed BALB/c mice (Endoxan 300 mg/kg). Immunosuppression was performed on the day of inoculation. The parasitemia of each mouse was measured daily using the matching method [13], beginning the 3rd day after infection. On day 3 post-infection, blood from infected mice was diluted in phosphate-buffered saline with 1% glucose (PSG 1%) and was injected into a further set of immunosuppressed BALB/c mice (2 × 10^6^ parasites per mouse, 22 mice) that were used to provide an infective feed to the tsetse flies.

### Infective feeding of tsetse flies

The tsetse flies were offered a single infective feed on day 3 or 4 post-inoculation of the mice when the parasitemia reached about 250 × 10^6^ parasites per milliliter. Sterile males of *G. f. fuscipes* were fed on the bellies of infected, anesthetized mice for 20 min, and only the engorged flies were then kept in the insectarium. All the engorged flies were then fed on sheep blood by in vitro membrane feeding 3 days a week for about 1 month.

### Dissection of tsetse flies

Midguts of all flies found dead were dissected daily except on weekends, from day 1 (D1) to day 17 (D17) post-infective meal. From D18 until D30 post-infective meal, all dead flies were dissected daily except on weekends, according to the method described by Penchenier and Itard [14] and the midgut and salivary glands examined. At D31–32 post-infective meal, the flies which were still alive were killed and immediately dissected to isolate the midgut and salivary glands. Before dissection, flies were starved for 72 h to reduce partially digested blood and thus facilitate observation of the trypanosomes in the midguts. All the midguts and salivary glands were examined for trypanosomes by phase contrast microscopy at 400 × magnification.

### Statistical analyses

To compare the success of infection by trypanosomes between treated and untreated flies with trypanocide we performed a Fisher’s exact test with the R-commander package [15, 16] for R [17].

## Results

**Fig. 1:**
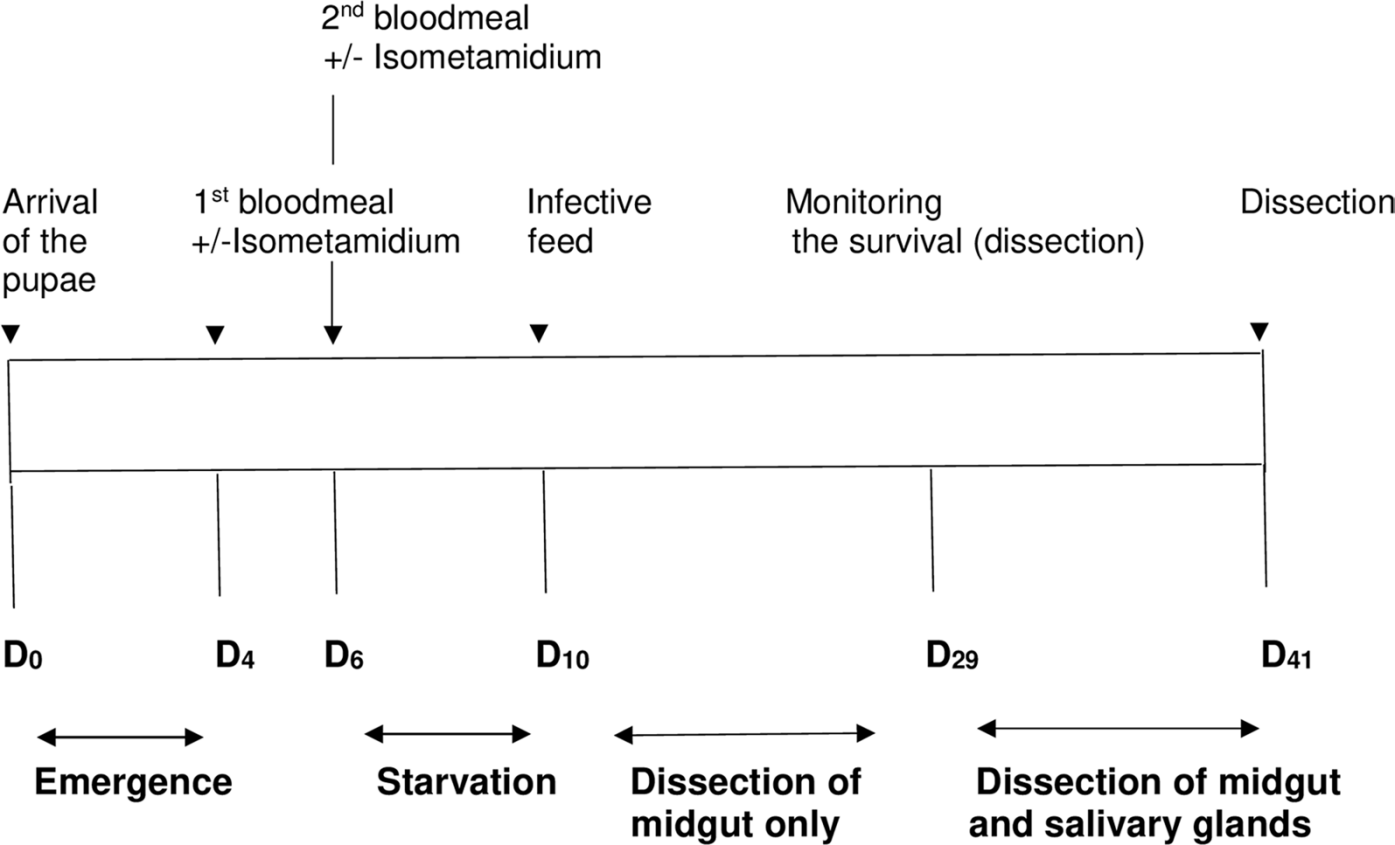
Experimental scheme used to evaluate the ability of sterile male *G. f. fuscipes* intended for use in the SIT program in Chad to acquire and transmit trypanosomes in the field

Of the irradiated pupae received from IAEA, 330 sterile males were fed on blood containing isometamidium chloride (batch 1) and 329 on blood alone (batch 2) 4 days post-reception of the pupae (see Fig. 1). By the time of the second feed (48 h later), with or without isometamidium chloride, 23.6% and 27% respectively of the sterile males from batch 1 and batch 2 were dead. Additional mortality (16.2% in batch 1 and 22% in batch 2) occurred between this second feed and the infective meal. The infective meal was offered to surviving sterile males from batch 1 (*n* = 211) and 2 (*n* = 187) 4 days post-second feed with or without trypanocide. More than 75% of the sterile males were engorged at the end of the infective meal with no difference between batch 1 and 2. A total of 160 sterile males from batch 1 and 145 from batch 2 thus received an infective meal.

Monitoring the survival of the flies for the duration of the experiment (from D0 to D31 post-infective meal) indicated > 75% mortality in the two batches.

A total of 144 sterile males from batch 1 and 130 from batch 2 were dissected between D3 and D32 post-infective meal (see Additional file 1: Table S1) because they either had died (from D3–D30) or were killed (on D31–32: end of the experiment). Of these, only six individuals [three from batch 1 (2.08%) and three from batch 2 (2.3%)] showed live trypanosomes in their midgut (see Table 1). Five of them contained large amounts of parasites in their midgut and one only a few trypanosomes. The proportion of flies hosting live parasites with or without prior treatment with trypanocide was not significantly different by Fisher’s exact test (*P*-value = 1).

**Table 1:** Microscopic observation of the midgut and salivary glands of six *G. f. fuscipes* for the presence of alive *T. b. brucei*

Day post-infective meal	Batch no.	Fly no.	Trypanosomes in the midgut	Trypanosomes in the salivary glands
3	1	15	+	ND
5	1	26	+	ND
7	1	29	+	ND
4	2	23	+	ND
31	2	234	+	−
32	2	258	+	−

Batch No.1 received isometamidium while batch No. 2 did not. + = alive trypanosomes and — = no trypanosomes found. ND: not done

Dead parasites could be observed on day 3 and 4 in the midgut of respectively two and one additional individuals from the two batches (see Additional file 1: Table S1), corresponding to non-established parasites. Whatever the batch, salivary glands infected with trypanosomes were not observed.

## Discussion

In this study, we evaluated the ability of sterile males of one strain of *G. f. fuscipes* intended for use in a SIT program in Chad to acquire and transmit trypanosomes under experimental conditions as follows. First, we applied a starvation period of 4 days before the infective meal to maximize the susceptibility of tsetse flies to trypanosome infection, as it has been reported that nutritional stress of tsetse at the time of the infective blood meal might enhance its ability to acquire trypanosomes [18]. Second, isometamidium chloride, a trypanocide with prophylactic action, was used (or not) prior to the infective meal to prevent development of trypanosomes in tsetse flies, as described in previous studies [11, 19, 20]. The trypanocide was given twice (at 2-day interval) to ensure that all the flies received at least one isometamidium meal [21]. Third, final dissection of the midgut and salivary glands occurred at D31–32 post-infective meal, a time lag usually used to maximize the likelihood of finding tsetse harboring mature infective metacyclic trypomastigotes [22–24]. In addition, all dead flies between D1 and D17 and D18 and D30 post-infective meal were also examined respectively for the presence of parasites in the midgut only or both in the midgut and salivary glands to increase the number of potentially positive flies.

In this study we faced a high mortality rate of the sterile male *G. f. fuscipes* with 23.6% and 27% dead from batch 1 and batch 2, respectively, between the two blood meals with or without isometamidium chloride. The additional 16.2% (batch 1) and 22% (batch 2) mortalities occurring before the infective meal were definitely due to the 4-day starvation period between the second meal with or without trypanocide and the infective meal. In addition to the mortality of the flies during the 10 days before the infective meal, there was > 75% mortality in each of the two batches during the infective process. Eventually, by subtracting the eliminated non-engorged flies, nearly 87% of the flies died between the first meal (when they were 4 days old) and the final dissection (when they were 41–42 days old). For comparison, the mortality of non-sterile and non-infected males *G. f. fuscipes* aged 41 days from the same colony, reared at the same time, was around 46% (data not shown). According to Vreysen et al. [25], average survival of non-sterile *G. f. fuscipes* males was about 57 days and about 49 days for 120 Gy-treated males under laboratory conditions. It is likely that storage at low temperature and subsequent transport of the pupae affected the survival of the *G. f. fuscipes* as has been demonstrated for *Glossina palpalis gambiensis* [26]. This however mimics the shipping protocol of pupae within an operational program.

In addition to the high mortality, the proportion of flies hosting live parasites was very low (2.08% with prior treatment with trypanocide and 2.3% without), and the parasites were observed in the midgut only. Midguts were found infected from the 3rd day post-infective blood meal. None of the dissected flies displayed trypanosomes in the salivary glands at D31–32 post-infective blood meal, indicating that the flies were unable to transmit the parasites.

From this limited experiment, the results suggest that the sterile males of this old-colonized strain of *G. f. fuscipes* could be refractory to trypanosome infection. On the contrary, a *G. p. gambiensis* colony, which had also been colonized for > 40 years and was challenged with the same *T. brucei* J10 WT strain, showed an infection rate in the midgut of 27% between D6 and D16 post-infective meal in our laboratory [27]. This hypothesis is strengthened by the fact that previous trials of experimental infections performed in our laboratory using two different strains of *T. brucei gambiense* and sterile males from the same colony of *G. f. fuscipes* resulted in no parasite observed in either midgut or salivary glands (data not shown).

Since the infection rate of non-irradiated males was not assessed, we cannot exclude that the refractoriness of such non-irradiated males is different from those that were irradiated. It would be interesting to implement the same experiment with non-irradiated males from the same strain. However, the goal was to assess the potential of released irradiated males to transmit HAT, and our results indicate that the risk appears to be neglectable with this strain. It needs to be emphasized that even in wild flies, the infection rate of tsetse flies with *T. b. gambiense* is very low, with mature infections of 0.1% reported in active HAT foci [28, 29].

In our experiment, isometamidium chloride used at a dose of 100 mg/l failed to prevent flies’ infection since, even if the prevalence of midgut infection was very low, no difference could be observed between the prevalence of flies treated or not by the trypanocide prior to the infective meal. For comparison, using the same concentration of isometamidium chloride, Van den Bossche et al. [11] observed a significant reduction of the fly’s immature (midgut) and mature (salivary glands) infection with *T. b. brucei* using a different trypanosome strain and *Glossina morsitans morsitans*. There is not much information about the mechanism by which isometamidium chloride reduces or inhibits the fly’s susceptibility to trypanosome infection. It is known that the trypanocide targets the kinetoplast of the trypanosome, accumulates there and linearizes the minicircles, which are essential in the editing process of the maxicircle genes [30–32]. Such disruption of the kinetoplast structure should impact the reproduction of the parasites leading to their death. We do not know how long the trypanocide can persist in the midgut of the flies to be available when they ingest the parasites.

Besides cyclical transmission by tsetse flies, one could consider the possibility of mechanical transmission of trypanosomes by released *G. f. fuscipes* sterile males. It has long been considered that mechanical transmission of trypanosomes by tsetse flies plays a much smaller role, if any, in the spread of HAT [33]. Experiments from Taylor [34] suggested that mechanical transmission must be extremely rare in the field where the contact between fly and human is rarely close enough to enable interrupted feeds to occur in periods as short as 30 min between subsequent feeds. Moreover the low parasitemia of *T. b. gambiense* usually observed in human blood [35] is a limiting factor for mechanical transmission.

## Conclusions

In conclusion, based on our laboratory observations in this limited experiment, the risk of cyclical transmission of *T. brucei* by sterile males from the strain of *G. f. fuscipes* tested appears small. Should another strain of *G. f. fuscipes* be selected for the SIT trial in Chad, a similar study should be repeated.

## Supplementary Information

**Additional file 1: Table S1.** Microscopic observation of the midgut and the salivary glands of 274 *G. f. fuscipes* (144 from batch 1 and 130 from batch 2) for the presence of *T. b. brucei*.

## Data Availability

Not applicable.
