# Supplementary material for: Vector competence of sterile male Glossina fuscipes fuscipes for Trypanosoma brucei brucei: implications for the implementation of the sterile insect technique in a sleeping sickness focus in Chad
Source: Parasit Vectors. 2023 Mar 22;16:111. doi: 10.1186/s13071-023-05721-4 (PMC10035118; doi:10.1186/s13071-023-05721-4)
Supplement: Supplementary file 1 — Additional file 1: Table S1. Microscopic observation of the midgut and the salivary glands of 274 G. f. fuscipes (144 from batch 1 and 130 from batch 2) for the presence of T. b. brucei. [file 13071_2023_5721_MOESM1_ESM.docx]

**Additional file 1: Table S1.** Microscopic observation of the midgut and the salivary glands of 274 *G. f. fuscipes* (144 from batch 1 and 130 from batch 2) for the presence of *T. b. brucei*.

Batch n°1 received isometamidium while batch n°2 did not. Missing: SG could not be dissected. ND: Not done. + : alive trypanosomes. - : no trypanosomes. †: dead trypanosomes.

| Day post-infective meal | Batch N° | Fly N° | Trypanosomes in the midgut | Trypanosomes in the salivary glands |
| --- | --- | --- | --- | --- |
| 3 | 1 | 12 | † | ND |
| 3 | 1 | 13 | - | ND |
| 3 | 1 | 14 | - | ND |
| 3 | 1 | 15 | + | ND |
| 4 | 1 | 19 | - | ND |
| 4 | 1 | 20 | † | ND |
| 4 | 1 | 21 | - | ND |
| 5 | 1 | 24 | - | ND |
| 5 | 1 | 25 | - | ND |
| 5 | 1 | 26 | + | ND |
| 7 | 1 | 27 | - | ND |
| 7 | 1 | 28 | - | ND |
| 7 | 1 | 29 | + | ND |
| 7 | 1 | 30 | - | ND |
| 7 | 1 | 31 | - | ND |
| 10 | 1 | 38 | - | ND |
| 10 | 1 | 39 | - | ND |
| 10 | 1 | 40 | - | ND |
| 10 | 1 | 41 | - | ND |
| 10 | 1 | 42 | - | ND |
| 10 | 1 | 43 | - | ND |
| 10 | 1 | 44 | - | ND |
| 10 | 1 | 45 | - | ND |
| 10 | 1 | 46 | - | ND |
| 10 | 1 | 48 | - | ND |
| 10 | 1 | 49 | - | ND |
| 10 | 1 | 50 | - | ND |
| 10 | 1 | 54 | - | ND |
| 10 | 1 | 55 | - | ND |
| 10 | 1 | 56 | - | ND |
| 10 | 1 | 57 | - | ND |
| 11 | 1 | 61 | - | ND |
| 11 | 1 | 62 | - | ND |
| 11 | 1 | 64 | - | ND |
| 11 | 1 | 65 | - | ND |
| 11 | 1 | 66 | - | ND |
| 11 | 1 | 67 | - | ND |
| 11 | 1 | 68 | - | ND |
| 12 | 1 | 83 | - | ND |
| 12 | 1 | 84 | - | ND |
| 12 | 1 | 85 | - | ND |
| 12 | 1 | 86 | - | ND |
| 12 | 1 | 87 | - | ND |
| 12 | 1 | 88 | - | ND |
| 13 | 1 | 89 | - | ND |
| 13 | 1 | 90 | - | ND |
| 13 | 1 | 91 | - | ND |
| 13 | 1 | 93 | - | ND |
| 14 | 1 | 96 | - | ND |
| 14 | 1 | 97 | - | ND |
| 14 | 1 | 98 | - | ND |
| 14 | 1 | 99 | - | ND |
| 17 | 1 | 100 | - | ND |
| 17 | 1 | 101 | - | ND |
| 17 | 1 | 102 | - | ND |
| 17 | 1 | 103 | - | ND |
| 17 | 1 | 104 | - | ND |
| 17 | 1 | 105 | - | ND |
| 17 | 1 | 106 | - | ND |
| 17 | 1 | 107 | - | ND |
| 17 | 1 | 108 | - | ND |
| 17 | 1 | 109 | - | ND |
| 17 | 1 | 110 | - | ND |
| 17 | 1 | 111 | - | ND |
| 17 | 1 | 112 | - | ND |
| 17 | 1 | 113 | - | ND |
| 17 | 1 | 114 | - | ND |
| 17 | 1 | 115 | - | ND |
| 17 | 1 | 116 | - | ND |
| 17 | 1 | 117 | - | ND |
| 17 | 1 | 118 | - | ND |
| 18 | 1 | 136 | - | ND |
| 18 | 1 | 137 | - | ND |
| 18 | 1 | 138 | - | ND |
| 18 | 1 | 139 | - | ND |
| 18 | 1 | 140 | - | ND |
| 18 | 1 | 141 | - | ND |
| 21 | 1 | 157 | - | - |
| 21 | 1 | 158 | - | - |
| 24 | 1 | 159 | - | - |
| 24 | 1 | 160 | - | - |
| 24 | 1 | 161 | - | missing |
| 24 | 1 | 162 | - | - |
| 24 | 1 | 163 | - | - |
| 24 | 1 | 164 | - | - |
| 24 | 1 | 165 | - | - |
| 24 | 1 | 166 | - | - |
| 24 | 1 | 167 | - | - |
| 24 | 1 | 168 | - | - |
| 24 | 1 | 170 | - | missing |
| 25 | 1 | 173 | - | - |
| 25 | 1 | 174 | - | - |
| 25 | 1 | 175 | - | - |
| 25 | 1 | 176 | - | - |
| 25 | 1 | 177 | - | - |
| 25 | 1 | 181 | - | - |
| 25 | 1 | 182 | - | - |
| 26 | 1 | 197 | - | - |
| 26 | 1 | 198 | - | - |
| 26 | 1 | 199 | - | - |
| 28 | 1 | 204 | - | - |
| 28 | 1 | 205 | - | - |
| 28 | 1 | 206 | - | - |
| 31 | 1 | 207 | - | - |
| 31 | 1 | 208 | - | - |
| 31 | 1 | 210 | - | - |
| 31 | 1 | 214 | - | - |
| 31 | 1 | 215 | - | - |
| 31 | 1 | 216 | - | - |
| 31 | 1 | 217 | - | - |
| 31 | 1 | 218 | - | - |
| 31 | 1 | 222 | - | - |
| 31 | 1 | 223 | - | - |
| 31 | 1 | 224 | - | - |
| 31 | 1 | 230 | - | - |
| 31 | 1 | 235 | - | - |
| 31 | 1 | 236 | - | - |
| 31 | 1 | 242 | - | - |
| 31 | 1 | 243 | - | - |
| 31 | 1 | 244 | - | - |
| 31 | 1 | 245 | - | - |
| 31 | 1 | 246 | - | - |
| 31 | 1 | 249 | - | - |
| 31 | 1 | 250 | - | - |
| 31 | 1 | 251 | - | - |
| 31 | 1 | 254 | - | - |
| 31 | 1 | 255 | - | - |
| 31 | 1 | 256 | - | - |
| 31 | 1 | 257 | - | - |
| 32 | 1 | 266 | - | - |
| 32 | 1 | 267 | - | - |
| 32 | 1 | 268 | - | - |
| 32 | 1 | 269 | - | - |
| 32 | 1 | 271 | - | - |
| 32 | 1 | 272 | - | - |
| 32 | 1 | 273 | - | - |
| 32 | 1 | 274 | - | - |
| 32 | 1 | 275 | - | - |
| 32 | 1 | 276 | - | - |
| 32 | 1 | 280 | - | - |
| 32 | 1 | 281 | - | - |
| 32 | 1 | 282 | - | - |
| 32 | 1 | 283 | - | - |
| 32 | 1 | 284 | - | - |
| 3 | 2 | 10 | † | ND |
| 3 | 2 | 11 | - | ND |
| 3 | 2 | 16 | - | ND |
| 3 | 2 | 17 | - | ND |
| 3 | 2 | 18 | - | ND |
| 4 | 2 | 22 | - | ND |
| 4 | 2 | 23 | + | ND |
| 7 | 2 | 32 | - | ND |
| 10 | 2 | 33 | - | ND |
| 10 | 2 | 34 | - | ND |
| 10 | 2 | 35 | - | ND |
| 10 | 2 | 36 | - | ND |
| 10 | 2 | 37 | - | ND |
| 10 | 2 | 47 | - | ND |
| 10 | 2 | 51 | - | ND |
| 10 | 2 | 52 | - | ND |
| 10 | 2 | 53 | - | ND |
| 10 | 2 | 58 | - | ND |
| 10 | 2 | 59 | - | ND |
| 10 | 2 | 60 | - | ND |
| 11 | 2 | 69 | - | ND |
| 11 | 2 | 70 | - | ND |
| 11 | 2 | 71 | - | ND |
| 11 | 2 | 72 | - | ND |
| 11 | 2 | 73 | - | ND |
| 11 | 2 | 74 | - | ND |
| 11 | 2 | 75 | - | ND |
| 11 | 2 | 76 | - | ND |
| 11 | 2 | 77 | - | ND |
| 11 | 2 | 78 | - | ND |
| 11 | 2 | 79 | - | ND |
| 11 | 2 | 80 | - | ND |
| 11 | 2 | 81 | - | ND |
| 11 | 2 | 82 | - | ND |
| 13 | 2 | 92 | - | ND |
| 13 | 2 | 94 | - | ND |
| 17 | 2 | 119 | - | ND |
| 17 | 2 | 120 | - | ND |
| 17 | 2 | 121 | - | ND |
| 17 | 2 | 122 | - | ND |
| 17 | 2 | 123 | - | ND |
| 17 | 2 | 124 | - | ND |
| 17 | 2 | 125 | - | ND |
| 17 | 2 | 126 | - | ND |
| 17 | 2 | 127 | - | ND |
| 17 | 2 | 128 | - | ND |
| 17 | 2 | 129 | - | ND |
| 17 | 2 | 130 | - | ND |
| 17 | 2 | 131 | - | ND |
| 17 | 2 | 132 | - | ND |
| 17 | 2 | 133 | - | ND |
| 17 | 2 | 134 | - | ND |
| 17 | 2 | 135 | - | ND |
| 18 | 2 | 142 | - | ND |
| 18 | 2 | 143 | - | ND |
| 18 | 2 | 144 | - | ND |
| 18 | 2 | 145 | - | ND |
| 18 | 2 | 146 | - | ND |
| 18 | 2 | 147 | - | ND |
| 18 | 2 | 148 | - | ND |
| 18 | 2 | 149 | - | ND |
| 18 | 2 | 150 | - | ND |
| 19 | 2 | 152 | - | missing |
| 19 | 2 | 153 | - | - |
| 19 | 2 | 154 | - | - |
| 20 | 2 | 155 | - | - |
| 20 | 2 | 156 | - | - |
| 24 | 2 | 169 | - | - |
| 24 | 2 | 171 | - | missing |
| 24 | 2 | 172 | - | missing |
| 25 | 2 | 178 | - | - |
| 25 | 2 | 179 | - | - |
| 25 | 2 | 180 | - | - |
| 25 | 2 | 183 | - | - |
| 25 | 2 | 184 | - | - |
| 25 | 2 | 185 | - | missing |
| 25 | 2 | 186 | - | - |
| 25 | 2 | 187 | - | missing |
| 25 | 2 | 188 | - | - |
| 25 | 2 | 189 | - | - |
| 26 | 2 | 192 | - | - |
| 26 | 2 | 193 | - | missing |
| 26 | 2 | 194 | - | missing |
| 26 | 2 | 195 | - | - |
| 26 | 2 | 196 | - | - |
| 27 | 2 | 200 | - | missing |
| 27 | 2 | 201 | - | - |
| 27 | 2 | 202 | - | missing |
| 27 | 2 | 203 | - | - |
| 31 | 2 | 209 | - | - |
| 31 | 2 | 211 | - | missing |
| 31 | 2 | 212 | - | - |
| 31 | 2 | 213 | - | - |
| 31 | 2 | 219 | - | - |
| 31 | 2 | 220 | - | - |
| 31 | 2 | 221 | - | - |
| 31 | 2 | 225 | - | - |
| 31 | 2 | 226 | - | - |
| 31 | 2 | 227 | - | - |
| 31 | 2 | 228 | - | - |
| 31 | 2 | 229 | - | - |
| 31 | 2 | 231 | - | - |
| 31 | 2 | 232 | - | - |
| 31 | 2 | 233 | - | - |
| 31 | 2 | 234 | + | - |
| 31 | 2 | 238 | - | - |
| 31 | 2 | 239 | - | - |
| 31 | 2 | 240 | - | - |
| 31 | 2 | 241 | - | - |
| 31 | 2 | 247 | - | - |
| 31 | 2 | 248 | - | - |
| 31 | 2 | 252 | - | - |
| 31 | 2 | 253 | - | - |
| 32 | 2 | 258 | + | - |
| 32 | 2 | 259 | - | - |
| 32 | 2 | 260 | - | - |
| 32 | 2 | 261 | - | - |
| 32 | 2 | 262 | - | - |
| 32 | 2 | 263 | - | - |
| 32 | 2 | 264 | - | - |
| 32 | 2 | 265 | - | - |
| 32 | 2 | 270 | - | - |
| 32 | 2 | 277 | - | - |
| 32 | 2 | 278 | - | - |
| 32 | 2 | 279 | - | - |
| 32 | 2 | 285 | - | - |
| 32 | 2 | 286 | - | - |
| 32 | 2 | 287 | - | - |
| 32 | 2 | 288 | - | - |
| 32 | 2 | 289 | - | - |
